# An Assessment of Scientific Evidence Relating to the Effect of Early Experience on the Risk of Human-Directed Aggression by Adult Dogs

**DOI:** 10.3390/ani13142329

**Published:** 2023-07-17

**Authors:** Ann Baslington-Davies, Helen Howell, Todd E. Hogue, Daniel S. Mills

**Affiliations:** 1 Department of Life Sciences, University of Lincoln, Lincoln LN6 7DL, UK; 2 School of Psychology, University of Lincoln, Lincoln LN6 7TS, UK; hhowell@lincoln.ac.uk (H.H.); thogue@lincoln.ac.uk (T.E.H.)

**Keywords:** aggression, bite, dog, fear, frustration, human–animal relationship, risk factor, systematic review

## Abstract

**Simple Summary:**

Dogs that bite or show aggression towards humans are a worldwide problem. Whilst many dog–human relationships are valuable and bring much happiness, when the relationship breaks down and results in an aggressive encounter, the consequences are, at best, unpleasant for all those involved. It is commonly believed that the early experiences of dogs may contribute to their behaviour as adults. We reviewed and synthesised the scientific evidence surrounding this belief in order to establish what it tells us about the importance of early life experiences with regard to minimising the possibility of dogs developing aggressive responses to humans as adult animals. We found evidence in support of the potential importance of the following risk factors: the source of the animal, the age at which it was rehomed, the reason for the acquisition, the experience level of the owner, the animal’s socialisation experiences, the consistent husbandry and management practices, the training, the sex ratio of the litter and the history of dogs that display aggression in the pedigree.

**Abstract:**

Human-directed aggression by domestic dogs is a major worldwide public health problem. The causes of aggression are complex, and research in this area often has to balance ecological validity with pragmatic controls; accordingly, it often does not meet the thresholds for quality typically used in reviews applying a classical “evidence-based” approach. Here, we propose a method of literature assessment that makes the “best use” of available evidence to identify and synthesise evidence relating to the most likely risk factors reported in the scientific literature. We used a systematic review process to initially identify relevant literature relating to potential early life experience (i.e., in the first six months of life) risk factors in the dog for human-directed aggression in the adult animal. Fourteen papers met our initial screening process and were subsequently analysed in detail, with data extracted and effect sizes calculated where possible. This highlighted the potential importance of the source of the animal, the age at which it was rehomed, the reason for the acquisition, the experience level of the owner, the animal’s socialisation experiences, the consistent husbandry and management practices, the training, the sex ratio of the litter and the history of dogs that display aggression in the pedigree as risk factors. Taken together, it seems that early experiences which limit the ability to develop effective coping strategies and routines may be particularly important. We provide guidance for the future standardised reporting of risk related to human-directed aggression by dogs to allow greater synthesis of the literature in the future.

## 1. Introduction

Human-directed aggression (HDA) by dogs is widely accepted as a significant risk to public health in many parts of the world [1], potentially leading to physical and psychological trauma for victims and welfare implications for dogs, who may be seized by the authorities for prolonged periods of time, rehomed or euthanised. There are economic costs associated with injury and treatment [2], possible legal proceedings and handling of dogs. The multi-disciplinary nature of HDA means that the issue has been explored with a focus on a range of factors, including breed differences [3], environment [4], role of the owner [5,6], role of the victim [7], and usefulness of prevention strategies [8] or legislation [9,10]. Professionals agree that the issue of dog bites is a complex one [11], but despite the relatively large volume of research on the topic, it seems that there is little confidence in the risk factors which may contribute to the development of HDA given the variable quality of related research [12]. Some risk factors are proximate, relating to the immediate antecedents of each incidence of HDA; other factors are more biologically ultimate [13], encapsulating the confluence of past experience reflected in the nature, personality, and behavioural tendencies of the individual dog. When considering the future risk for HDA, there is specific value to considering these historical features that contributed to the development of the instance of aggressive behaviour. Systematic reviews generally seek to identify relevant literature, objectively appraise the quality of studies and then include or exclude items based on reliability and relevance [14]. These screening processes have generally been developed to assess the quality of single interventions within the medical field and do not translate well into the assessment of risk in human–animal interactions, where the effects are multi-factorial and often very complex, with variation between situations [15]. By applying a modified version of the Scottish Intercollegiate Guidelines Network (SIGN) levels of evidence tool [16], which added analytical cross-sectional studies to level 2—the original tool can be seen at https://www.sign.ac.uk/assets/sign_grading_system_1999_2012.pdf (accessed on 13 July 2023), Newman [12], in her systematic review of HDA literature, sought to objectively appraise the quality of studies and therefore utilised a pre-determined threshold for the determination of useful evidence, although, through modification, she sought to minimise the effects of excluding potentially useful material based solely on the supposed quality of evidence.

Nevertheless, Newman [12] still determined that only eight papers (from a subset of 164 studies that had an appropriate design and comparator group) should be taken through to the final analysis. She concluded there was no robust evidence for any of the factors she examined and identified a limited number of studies that provided a moderate level of somewhat conflicting evidence. The risk factors evidenced were neutering, the presence of young people in the home, the heritability of aggression and the management of the dog by its owners. It was acknowledged that this by no means indicated that other important risk factors did not exist, only that robust evidence for them was lacking.

It is recognised that the rejection of much of the corpus of knowledge due to setting very high standards for individual studies is overly limiting and may be problematic when considering matters of substantial health or economic significance. An alternative is to use a more bespoke assessment of the data integrity, examining the existing research to identify meaningful evidenced patterns within the data [15]. All research is subject to error, and individual studies may have methodological weaknesses and confounds. However, if diverse studies with different designs, each with their own limitations, consistently point to a similar finding, then these factors deserve more careful consideration and assessment, especially when a field lacks much definitive research [15]. Such an approach helps to rationalise future, more targeted, higher-quality research on matters of potential importance with adequate controls. However, this does not overcome the potential problem of bias relating to the areas which may have attracted the most research attention [15]. The amount of research focusing on a specific risk factor should not be confused with its practical importance nor divert consideration away from other possible factors. Only by reviewing what has been considered and the ensuing results can the limits to the research questions asked in a given field be established. Researchers and stakeholders need to be aware of the dangers of honing in on the results of one study and overgeneralising it. This is particularly the case for qualitative rather than quantitative studies, where providing a rich, contextualised understanding of an issue is more important than generalising the results [17]. The complex idiographic nature of incidences of HDA requires an appreciation of the variance between individuals in a group. At the same time, there is much to be gained from a tentative and critical synthesis of the available research findings into such a socially important topic as HDA.

There are undoubtedly many factors that contribute to the expression of HDA, and oversimplification of the concept of cause by emphasising the importance of one type of factor over another is clearly unwise [18]. This is a problem well-illustrated by the problems associated with breed-specific legislation [10,19,20]. The role played by the owner and their culture, the circumstances at the time of any biting incident, the health of the dog and the role of the victim are all important considerations in relation to the immediate expression of this problem. However, this does not undermine the value of identifying meaningful, general and/or consistent non-proximate risk factors from an ontogenetic and pre-ontogenetic perspective [21]. Less proximate factors include the effect of early life experiences. Although these may not always be amenable to management to control the risk of a problem within a given individual, they are nonetheless important to understand the broader nature of HDA, which is essential to the formulation of the problem and implementation of the future reduction of risk and prevention strategies.

These factors interact to affect the likelihood of a dog displaying HDA. Dogs, in common with many other species, have sensitive developmental periods when they are particularly receptive to the consequences of certain experiences and stimuli [22,23]. The carryover effects of these early life experiences impact the likelihood of specific behaviours in certain contexts [24,25]. These behaviours can be stimulus-specific or more general predispositions reflected in the animal’s temperament, which is relatively stable by the time an animal is fully mature [26]. Different breeds of dogs mature at different rates, but by 12 months old, most dogs are considered adults and have a relatively well-defined character in many regards, even if they are not fully socially mature. By this age, companion dogs are usually housed or living in places occupied by people, and if, as a result of earlier life experiences, the dog has learned to use aggressive behaviour towards people, this may present a risk to both dog and human. It is accepted that for some breeds, puberty may begin within this six-month window, and this could be a factor worthy of consideration. However, no literature relating to puberty as a risk factor for aggressive behaviour within the first six months of life was found in this search. Aggressive behaviour from a fully developed adult dog is potentially far more damaging than similar behaviour from a physically smaller and developmentally less mature puppy.

The aim of this study was to identify and evaluate the risk factors described in the scientific literature relating to the early life experiences of a dog on the subsequent risk of HDA as an adult. Accordingly, methodological standards of evidence required for consideration were adjusted to allow for maximal identification of potential risk factors considered in the scientific literature, with subsequent evaluation of the evidence being based on the totality of studies rather than on each individual study [15]. The focus was on the risk of the likelihood of HDA rather than its severity, as severity may have different risk factors or present a quantitatively different level of risk for a given risk factor.

## 2. Materials and Methods

### 2.1. Study Design

This review aimed to update and expand upon the previous review of Newman et al. [27], published as Newman [12], which examined the evidence base of risk factors for HDA using stringent evidence-based criteria for assessment inclusion criteria. In the current study, Preferred Reporting of Items of Systematic Reviews and Meta-Analyses (PRISMA) guidelines were followed [28]. Broad inclusion criteria were adopted in the current review, as it was anticipated that the quality of evidence would be variable. Being able to identify the breadth of risk factors covered in the literature, regardless of quality, was a secondary goal of the current work, as well as undertaking a detailed assessment of the quality of the evidence. The intention was to utilise, as far as possible, the available research information rather than exclude research information based on a strict criterion related to research methodology, as was achieved by Newman [12].

The protocol received ethical approval from the delegated authority of the University of Lincoln (UoL2021_6411).

### 2.2. Search Strategy

Search terms based on those used by Newman [12] formed the basis of the current search. These terms were piloted and refined (to exclude rabies) on the intended databases to improve the amount of relevant literature returned. The terms used in the search were (dog OR dogs OR canine) AND (bite OR bites OR aggression OR attack) NOT (rabies).

### 2.3. Systematic Review Protocol

The initial inclusion criteria for article selection were the following:

1. Articles written in English;
2. Articles accessible via direct download from the University library or through contact with the authors;
3. Articles published from 2010 to the end of February 2021.

### 2.4. Data Sources

Between 5 August 2020 and 28 February 2021, searches were conducted in the following electronic databases: Google Scholar, Science Direct, Scopus and PsychINFO. Grey literature searches were conducted in Opengrey and Proquest. 

The outputs from this search were then supplemented with articles from before 2010 taken from the full list of the results of Newman’s initial search [12], using the same search terms. This work included publications from 1960 to 31 December 2010.

### 2.5. Initial Appraisal

Potentially relevant literature was imported into EndNote. Duplicates were eliminated. All remaining papers then underwent initial appraisal for potential suitability based primarily on the title and abstract. Two researchers worked together to agree on which articles should be retained. Articles were excluded if they were not primary research or if they related to other aspects of human-directed dog aggression, such as interventions for management. Included articles were therefore focused only on the potential risk factors for HDA.

### 2.6. Review and Analysis of Data

Each paper was analysed to identify the dependent variable (i.e., some form of HDA) and the specific risk factors of interest (independent variables), for example, where the dog was acquired from. To quantify effect sizes, a clear comparison or control group needed to be identified (i.e., a reference variable). The average effect size/odds ratios with confidence intervals were extracted when available. If this information was not directly provided, it was calculated where possible from the information provided in the paper using the Select Statistics odds ratio/confidence interval calculator [29]. The resulting summary data was then consolidated into tables to allow comparison across studies (see Results section for full details).

The corresponding authors for all papers included for analysis were e-mailed with a request for copies of raw data and, where appropriate, the questionnaires used. Four replied [30,31,32,33], and three were able to provide some data [30,31,32]. Research by Westgarth, Reevell and Barclay [32] was subsequently excluded as it concerned dogs with multiple behavioural issues and aggression was not identified as a discrete outcome (dependent variable). The information provided by O’Sullivan et al. [30] (an example of the questionnaire used) and Foyer et al. [31] (an example of the raw data) could not be used to calculate odds ratios and/or confidence intervals as the information contained was not relevant to being able to make the related calculations.

For the purposes of a systematic review, a system for grading evidence is usually adopted. This allows for a clear distinction between studies deemed to be of an acceptable standard and those which are not; however, this type of censoring might lose valuable information. The OCEBM (2009) levels of evidence are frequently used as the gold standard for setting such thresholds [34]. However, this was designed to assist medical practitioners in assessing the quality of evidence surrounding treatment options and not determining if research is of any value. The OCEBM system is also of limited value when applied to studies that are non-experimental in nature. These challenges are explored by Newman [12], who decided to utilise a modified form of SIGN as a result [12,16]. Despite this enabling the inclusion of additional material, the thresholds applied for inclusion meant that only eight papers were included in the final analysis. For the current review, evidence-based medicine thresholds were not applied on the basis of study design for the purposes of inclusion, but each paper was assessed for its merits and limitations within the constraints for that kind of study, e.g., retrospective analysis. The limitations of each study were carefully described and considered; studies were excluded only if they did not meet the initial search criteria previously outlined and did not explore risk factors for sub-adult dogs. This provided a larger pool of information and enabled the identification of a broader range of factors but also introduced a potential risk through the inclusion of studies of much more variable quality. This risk can be addressed by specific acknowledgement and critical appraisal of the content and design of these studies to ensure they are kept in context.

## 3. Results

### 3.1. Publications Retrieved

The initial search identified 30,233 papers. Following the initial appraisal, 29,529 were eliminated, leaving 704 papers (2.33% of returned papers). The reference lists of narrative and systematic reviews found during the initial appraisal were searched for papers not previously identified, and 10 papers were subsequently added to the initial database. A further 85 papers were added from Newman [27], and duplicates (n = 286) were then removed, giving a total of 513 papers. A further 339 were excluded on the basis of a closer examination of their title, primarily due to a lack of risk factors being identified in the work. Of the remaining 174 articles, the abstract was read, and a further 147 were excluded as they were not obviously associated with risk factors in the early life experiences of the dog (i.e., occurring within the first six months). The 27 remaining papers were read in full. In total, eight were excluded from the analysis as they considered risk factors for aggressive behaviour in young dogs rather than adults. One other was excluded as it was only published as a conference abstract. The study by Westgarth et al. [32] was also excluded at this time, as it concerned dogs with multiple behavioural issues; however, aggression was not identified as a discrete outcome. This left 14 papers for analysis, of which 11 considered the risk in dogs over 12 months of age, and 3 stated that the “at risk” population were adults.

Figure S1 (Supplementary Materials) summarises the screening and appraisal process used, in line with the Preferred Reporting Items for Systematic Reviews and Meta-Analyses guidelines [28].

### 3.2. Factors Relating to Early Experiences

The papers considered in the assessment of the risk posed by early life experiences on human-directed aggressive behaviour in adult dogs are listed in Table 1. In some papers, authors stated that the dogs were adults but not explicitly that they were 12 months old or more; these were included. 

Table 2, Table 3, Table 4 and Table 5 summarise the information relating to the risk factors identified from a bivariate analysis in the 14 papers of interest. This includes details of the average effect size/odds ratios with confidence intervals when it was available or calculated on the basis of the available data. Types of aggression considered are general HDA (Table 2), owner-directed aggressive behaviour (Table 3), aggressive behaviour towards family members and familiar people (Table 4), and towards strangers and unfamiliar people (Table 5).

In summary, general HDA (rather than aggressive behaviour towards a specific subset of humans, such as owners or strangers) was only found to be really noticeable in dogs that had been raised with very limited human contact. The dogs in that study [43] were livestock-guarding dogs in remote areas and were not intended to be companion animals. Attendance at puppy class and source of acquisition were not shown to make any difference to the likelihood of general HDA [30,44]. Two papers, both from the same authors [31,38] and looking at military working dogs, showed higher levels of aggressive behaviour for dogs born at certain times of the year, although the results conflicted. An examination of training methods used [40] showed that using positive reinforcement methods only or using consistent punishment and positive reinforcement decreased the risk of aggression, whereas using inconsistent punishment alongside positive reinforcement nearly doubled the likelihood of dogs developing aggressive responses. The authors in this paper generalised aggression to all people (familiar and unfamiliar) and to other dogs.

In summary, dogs acquired from pet shops were shown to be, on average, up to 2.4 times more likely to show owner-directed aggressive behaviour compared to dogs from breeders [33], and if acquired from a hobby breeder [36], they were, on average, up to 1.3 times more likely to develop owner-directed aggression. Dogs whose availability was advertised in a newspaper or posted advert [36] were up to 1.7 times more likely, on average, to show owner-directed aggressive behaviour compared to dogs found by word of mouth or by contacting a breeder. Feeding a dog from the table [40] was associated with a fivefold increase in the risk of owner-directed aggressive behaviour (but see the Discussion section for the full rationale).

Dogs acquired for the purposes of hunting, rather than as pets or guard dogs, were slightly less likely to develop owner-directed aggressive behaviour [36], as were puppies being raised by owners with previous experience in raising puppies [41]. Growing up with other dogs in the home also reduced the likelihood of owner-directed aggressive behaviour [41].

Lack of training and being allowed to sleep in the owner’s bed did not show a significant association with aggressive behaviour towards the owner [36].

In summary, dogs from a non-domestic maternal background that were not showing avoidance behaviour or aggressive behaviour to unfamiliar people were, on average, up to 1.43 times more likely to show aggressive behaviour to familiar people than those from a domestic maternal background [24]. Dogs homed before 7 weeks of age were, on average, seven times more likely to show aggressive behaviour to family members than those homed after 7 weeks old [40]. When considering training methods, any use of positive punishment at all was significant compared to owners who did not use positive punishment. Dogs trained by use of a combination of positive reinforcement and positive punishment produced the highest mean aggression score [37].

When compared to dogs from a domestic environment, dogs from a non-domestic environment were, on average, up to 17 times more likely to show aggressive behaviour to unfamiliar people only when away from home, nearly three times more likely to show aggressive behaviour to unfamiliar people only in the home, and 1.7 times more likely to show aggressive behaviour to unfamiliar people both in and away from home [24]. Dogs from a non-domestic maternal environment that did not also show avoidance behaviour were not much more likely to show aggression to unfamiliar people than those from a domestic environment [24].

With respect to homing age, dogs homed at 6–7 weeks were not more likely to show aggressive behaviour to unfamiliar people either inside or outside the home, compared to dogs homed at 8–16 weeks [42]. Dogs homed at 8 weeks were not more likely to show aggressive behaviour to unfamiliar people inside the home but were over 6 times more likely to do so outside the home compared to dogs homed at 6–7 weeks or 9–16 weeks [42]. Dogs homed at 9–12 weeks were slightly less likely to show aggressive behaviour to unfamiliar people either inside or outside the home compared to dogs homed at other ages. When homed at 13–16 weeks, dogs were twice as likely to show aggressive behaviour to unfamiliar people inside the home compared to dogs homed at other ages but not more likely to show aggressive behaviour to unfamiliar people outside the home [42].

Puppies raised by owners with previous experience in raising or owning puppies were less likely to demonstrate aggressive behaviour to strangers or unfamiliar people [41].

No difference was found with regard to stranger-directed aggressive behaviour between dogs that had socialised with people through attendance at a puppy class and those that had not [44]. Dogs that had been threatened by another dog as a puppy were slightly more likely to react aggressively to strangers [41].

Dogs that had been fed from the table in the two months preceding an aggressive incident were 3 times more likely to have shown aggressive behaviour to strangers previously [30].

Dogs with a previous history of aggressive behaviour (i.e., more than one aggressive incident) were found to behave more aggressively towards strangers if they had been subjected to physical or verbal punishment (on average 5 times more likely), allowed to win games of tug or initiate play (3 times more likely), and display a differing level of obedience according to location and person in charge (on average 3 and 4.2 times more likely) [30]. Dogs that showed fearful reactions were, on average, 5 times more likely to show stranger-directed aggressive behaviour, 6 times more likely to display aggressive behaviour to strangers if they also displayed other problem behaviours in the presence of family members, 4 times more likely if they displayed three or more excessive behaviours and over 6 times more likely if they were not trusted around children. These results came from a paper comparing dogs that had only bitten once with dogs that had a sustained history of aggressive behaviour [30]; this is considered more fully in the Discussion section. The same paper found that dogs with a multiple bite history that did not respond to the ‘sit’ command were slightly less likely to show stranger-directed aggressive behaviour when compared to dogs with only a single bite history.

In addition to the information in the above table, a range of non-significant risk factors were also identified:

No significant associations between homing age and HDA to familiar people were found [42].

Parity, birthweight, growth weight and litter size were found not to be significantly associated with HDA [31].

Dogs who had attended puppy classes were no more likely to show aggressive behaviour than non-attending litter mates in a range of circumstances, including possession of resources, being handled/groomed, being disturbed when asleep, being reached for or loomed over and being territorial [35].

Variability in dog obedience according to the person or location; presence of behavioural problems when family members present; three or more behaviours considered to be excessive; dog not trusted with children; and dog being allowed onto the furniture in the 2 months preceding the bite incident were not taken forward for multivariate analysis by O’Sullivan et al. [30].

## 4. Discussion

From the 14 papers considered, the risk factors investigated in the literature can be thematically divided into the following categories: source of acquisition (home breeder, pet shop, rescue centre, commercial breeder); age at which the dog was rehomed; in theory this should be the age at which it left its mother—however, in some cases, a dog traded by a third party may have left its mother some weeks in advance of being rehomed (it was not possible to determine if this was the case from the papers examined); reason the dog was acquired (pet or working); experience level of the owner (how many dogs had they previously owned or fostered); socialisation experiences (socialised with children or other dogs, negative experiences during socialisation); husbandry and management (being fed from the table, sleeping on the owner’s bed, use of verbal or physical punishment, management of play behaviour); training (method and amount of training); sex ratio of litter; and history of dogs displaying aggressive behaviour in the pedigree. Notable for their absence include consideration of the following: amount and type of exercise/rest, often considered by professionals to be important aspects of limiting or mitigating aggressive responses [45]; health (particularly related to pain), which has been a focus of recent research [46,47]; and specific information about interactions with children, who are more likely to be the victims of dog bites than adults [48]. Extensive research has examined the medical implications of dog bites in children [49,50], and some has focussed on the benefits of prevention and education programmes [51,52], but research looking at the ways in which bites to children can be mitigated by focussing on the young dog are lacking. Before discussing these factors in detail, it is important to recognise the impact on the findings given the variability in the definition of HDA used by authors and comparison reference populations being used. 

It has previously been recognised that a major challenge to scientific progress in this field relates to the definition of aggression and aggressive behaviour [11], and the current work reinforces this issue. There was both variability and inconsistency in the definitions of human-directed aggressive behaviour used in the studies considered here. Some, e.g., Blackwell et al. [37], provided no definition of aggressive behaviour in the paper; others, e.g., Marion et al. [43], defined aggressive behaviour in terms of barking, lunging or biting; while McMillan et al. [39] and González-Martínez et al. [44] used the CBARQ questionnaire to get owners to rate their dogs’ behaviour in certain situations and defined aggressive behaviour along a continuum from ‘growling/barking, baring teeth, snaps, bites or attempts to bite’. Six further papers included similarly wide-ranging behavioural definitions such as ‘growl, bite, snap, lunge, snarl’ [24,31,33,38,40,41] but sometimes with no clear elaboration on the definition of these behaviours. Jokinen et al. [42] defined it as a ‘growl, snap, bite or attempt to bite’ and thus would presumably exclude lunging and snarling dogs unlike the former authors. At the other end of the spectrum, Martin [35] provided body language-related definitions to owners, including a tense body escalating to biting, which would considerably broaden the scope of what constitutes aggressive behaviour compared to the majority of studies, where early signals of discomfort, such as tension, are not generally included. By contrast Reisner et al. [36] differentiated between owner and stranger-directed aggressive behaviour but defined these differently: aggressive behaviour towards owners involved a growl, snap or bite but towards strangers only a bite was considered. Thus, it must be recognised that different studies are examining the risk factors for different outcomes, even if they are all classified as human-directed aggressive behaviour. This point is further emphasised by the variability in the human targets for the aggressive behaviour considered. For example, Pirrone et al. [33] considered aggressive behaviour towards owners, but not strangers; which might represent a distinct sub-population within the broader class of human-directed aggressive behaviour. 

Similar issues arose with the definition of the control groups; for example, Le Brech et al. [40] also focused on aggressive behaviour towards owners but included dogs displaying aggressive behaviour directed at strangers in the control group, meaning the results relate to risk factors that might discriminate owner-directed aggression from that directed towards strangers. By contrast, Appleby et al. [24] and Serpell and Duffy [41] considered aggressive behaviour towards owners, strangers and known and unknown dogs, i.e., risk factors common to all of these scenarios.

The selection of adequate control groups for retrospective studies is a major challenge since very few dogs have no behavioural issues [53]. For a control with no behaviour problems to be valid, then dogs displaying aggressive behaviour would need to have no problems other than aggression; this is possibly an unusual scenario given the emotional basis of aggressive behaviour [11]. Several authors appeared to recognise this issue and accepted associated limitations or used designs that circumvented this issue. For example, González-Martínez et al. [44] used a cohort design to evaluate the effects of puppy classes on adult behaviour; they paired dogs that had not attended classes with similar-aged dogs that had; likewise, Martin [35], who also looked at the effect of puppy classes, used littermates where one had been to classes, and one had not to form matched cohorts. By contrast, Appleby et al. [24], who also looked at the effect of early life experiences, used a control group that included dogs with behavioural issues associated with a lack of control and attention seeking. They argued that these problems relate to owner reinforcement, and so would not be substantially affected by the variables of interest to them, i.e., the dog’s maternal environment, the age it was acquired or the type of stimuli encountered early in life (the variables of primary interest). Other control groups were perhaps more questionable in terms of their value, such as dogs known to have bitten on only one occasion versus dogs with no bite history [30]; this was justified on the basis of an assumption that a single bite history is probably just an accident. Le Brech et al. [40] focused on owner-related aggressive behaviour and thus included some dogs that had shown aggressive behaviour to strangers but not to owners in their control group. These differences clearly reflect the specific aims of the studies and thus limit the extent to which more general conclusions can be drawn on the subject of the risk of human-directed aggression. However, this may not always be appreciated, and there is a risk of overgeneralisation. This is evident from legislation that prohibits or places severe restrictions on the ownership of entire breeds, irrespective of the behaviour of individual dogs. Regrettably, such a naïve approach explains why the number of hospital admissions for dog bites has increased despite the prohibition of certain types of dogs in order to supposedly reduce this risk [54].

One way to potentially address this limitation in a specific study is to examine the evidence of consistency in risk factors observed across different studies and study methodologies. For example, three studies noted that dogs acquired from non-domestic environments were more likely to display aggressive behaviour towards humans than those acquired from sources where greater early socialisation experiences were more likely to have been available [24,33,39]. Whilst this approach may reduce the ability to specify precise variables, i.e., it is impossible to identify if any specific aspect of socialisation is more important in reducing aggressive behaviour, this broader classification of risk is useful and is consistent with the generally held view that early familiarisation with household environments is important to puppy development. Along a similar theme, the lack of early socialisation experiences with people, dogs and children was explored by three researchers [30,41,43], and all found that dogs were more likely to display HDA if socialisation with any or all of these was lacking. This indicates that both the early physical and social environment impact the risk of HDA in adult dogs. 

Even in cases where the primary objective of the research differed substantially, important commonalities in the results can be noted. For example, when looking at homing age, Le Brech et al. [40] found that dogs homed before 8 weeks of age were 7 times more likely to display aggression to family members than those homed at 8 weeks old or more, whilst Jokinen et al. [42] found that aggressive behaviour to unfamiliar people was slightly more prevalent in dogs that had been homed before 8 weeks. Although the targets for HDA differed between these two pieces of research, the notion that early rehoming (i.e., before 8 weeks) can be a factor in HDA was consistent in both studies. These results should not be considered conclusive, but they do show scientific consensus on relevant risk factors, on which future studies can build.

The work of Newman [12] highlighted concern over the quality of the studies from an evidence-based medicine perspective, with only 8 out of 164 full articles that were assessed for methodological quality considered of sufficient quality to be included in her synthesis. In addition, it should be noted that the strength of evidence is also affected by the type of analysis undertaken. In the current study, 10 of the 14 papers examined the statistical significance of the risk factors using simple bivariate associations (i.e., without controlling for potential confounds), whilst the remaining 4 used a multivariate analysis [30,38,39,42]. The most common factors controlled for in these models were the following: other dogs in the household [39]; role of the dog (working or pet) [39]; gender of the dog [39]; bodyweight of the dog [39]; neuter status [39]; age of rehoming [42]; and socialisation [30]. Studies using multivariate analysis potentially provide more robust evidence as they acknowledge to some extent, the multi-factorial nature of HDA in adult dogs and so reduce the risk of spurious confounds. This becomes increasingly important as a more reliable evidence base is built. However, when knowledge of a phenomenon is very limited, the simpler bivariate analysis may be of value in formulating hypotheses to prioritise for more rigorous assessment. Caution is warranted when drawing conclusions from bivariate analysis to reduce the risk of applying inappropriate or ineffective interventions when so much remains unknown, but it suggests the evidence should not be simply dismissed since scientific progress is incremental and should always be accompanied by critical reflection at each stage [15].

With the caveats described above in mind, given the limited quality of the studies from an evidence-based perspective, further value from published studies can be extracted by considering the magnitude of the risks identified rather than simply their statistical significance [15]. The issues identified above with regard to control populations and definition of outcome preclude any form of reliable meta-analysis, but careful examination of effect sizes on an individual study basis is worthwhile. Where it was possible to examine odds ratios for HDA, they ranged from a mean reduction of 0.2 for socialisation with other dogs and for not responding to the ‘sit’ command [30] to an increase of 11.6 for the use of verbal or physical punishment [30]. Overall, considering the issues of consistency across studies, magnitude of effect, and study designs that control for confounds, it seems that there is most robust evidence for the importance of the following broad factors:

Acquisition-related factors;
Management and husbandry;
Early litter experience, including composition.

These are considered in more detail in the following sections.

### 4.1. Acquisition-Related Factors

Acquisition-related factors include aspects such as what sort of environment the puppy was born into, consideration of the early socialisation experiences available at this time, how long the puppy remained with its dam and siblings before being rehomed and the role the prospective owner expects the dog to fulfil in the future. In the current review, the age of rehoming was investigated by Jokinen et al. [42] and Le Brech et al. [40], and both found that puppies rehomed between 6–8 weeks old were less likely to develop HDA, despite differences in methodology between the two papers. O’Sullivan et al. [30] also noted in a survey of 100 dogs that had bitten a person that nearly 60% were acquired at 12 or more weeks of age, suggesting that dogs acquired for the first time at this later age may be predisposed to develop HDA.

The development of behavioural problems including, but not limited to, aggressive behaviour, has been linked to rehoming puppies outside of a particular age window, i.e., before 6 weeks or after 10 weeks, and the nature of the environment they are raised in during their early weeks [25]. The optimal age for removing a puppy from the dam and littermates and placing it in its new home has been considered as 6–8 weeks for many years, based on an interpretation of the result of early isolation experiments of researchers such as Scott and Fuller [55]. This has been used to suggest that allowing puppies to stay with their dam and littermates until 6–8 weeks old will allow them to gain important dog-related socialisation experience through interactions with other dogs from their litter home whilst allowing sufficient time for socialisation and habituation with commonplace household objects and people in the new home. Puppies acquired from environments lacking in social and/or environmental stimuli are also reported to be more likely [25,56] than those bred in a home environment to display fearfulness and have medical problems, both of which may increase the risk of aggressive behaviours [46,47,57].

Five studies focused on the source of acquisition [24,30,33,36,39], all suggest that there is an increased likelihood of HDA presenting in adult dogs that had been obtained from environments with more limited socialisation/habituation opportunities compared to those acquired from those who potentially had the dogs living in the house. Potentially suboptimal environments included pet shops and sourcing from commercial suppliers [33,39], from a newspaper advert [36] or from a non-residential part of the breeder’s home [24]. Sub-optimal environments are quite probably stressful for the mother, and this can have a deleterious effect on the future behavioural development of the puppy, but this factor was not discussed in depth by any of the papers reviewed. The consensus between researchers, despite differing methodologies, lends weight to the importance of the early rearing environment, with average odds ratios for increased aggressive behaviour towards humans as an adult ranging from 1.09 to 17.86. In contrast, Blackwell et al. [37] found that the origin of the dog (breeder, home bred or rescue shelter) had no effect on aggressive behaviour, although the main focus of this paper was attendance at training classes, not the source of acquisition. O’Sullivan et al. [30] also did not find any difference in HDA based on source, whether it was a rescue kennel compared to a breeder or a private owner. The meaning of conflicting results can be harder to evaluate in terms of the hypothesis being tested since a lack of effect is not the same as there being no effect. In contrast, Casey et al. [58] found that dogs from rescue centres were 2.6 times more likely to display HDA and Flint et al. [59] noted that dogs acquired from a shelter were 1.28 times more likely to display aggressive behaviour towards strangers than dogs acquired from other sources. Further evidence of the need for caution in evaluating the importance of the results of O’Sullivan et al. [30] and Blackwell et al. [37] comes from Diesel et al. [60], who noted that over 10% of dogs relinquished to Dog’s Trust centres in the UK (a charity that does not euthanise healthy dogs) were surrendered due to aggressive behaviour. It is also worth noting that O’Sullivan et al. [30] and Blackwell et al. [37] do not provide information on how old the dogs in their studies were when entering rescue, although Carter and Martin [61] identified that dogs under a year old made up the smallest group of dogs entering UK rescues, so it may be that those dogs included in O’Sullivan et al. [30] and Blackwell et al. [37] were not puppies when they entered rescue and may have previously been subject to a domestic rearing environment. Alternatively, aggressive individuals may have been culled from the population. 

### 4.2. Management and Husbandry

The second common theme to emerge in the current review relates to the management and husbandry of the puppy once it has moved to its new home. This theme broadly includes on-going socialisation/habituation experiences, interactions with other dogs, training, and day-to-day behavioural management. 

Researchers considered different aspects of this experience, for example, exposure to dogs [30,41], children [30] or people in general [41,43,44], and all reported that dogs lacking in socialisation (in general, but see the specific effect reported by O’Sullivan et al. [30] described below) were more likely to display HDA despite the different focus of each piece of research. The contexts in which socialisation was explored differed widely. Marion et al. [43] looked at livestock-guarding dogs and their early experiences with humans. Dogs that were raised with very limited human contact were five times more likely to exhibit HDA than dogs raised with more human contact. In contrast, Serpell and Duffy [41] considered the influence of the puppy-raising environment on potential Guide Dogs, where puppy raisers are routinely supported by the Guide Dogs Association in the socialisation process. Those dogs raised with another dog displayed significantly lower levels of aggression towards household members than puppies raised on their own. The authors suggest this could be due to the experience of people who already own dogs and to the socialising effects of another dog in the house. 

A perceived association between HDA and dog–dog aggression might arise because intervening between dogs that are fighting can result in injury to a person. As such, it may be that dogs not showing aggression to conspecifics may be less likely to accidentally bite a person. Although O’Sullivan et al. [30] reported a general positive effect of socialisation, dogs that were not socialised with and were aggressive towards other dogs showed decreased odds of HDA (OR = 0.2). Possible explanations include that people who know their dogs are aggressive towards other dogs actively avoid contact with other dogs and owners when on walks. They may also exercise more caution with their dogs, not just during potential interactions with other dogs but also with people. Nevertheless, the result does not accord with the findings of other researchers [41,62]; nonetheless, the potential association needs to be treated with caution until further evidence is available. It is worth noting that puppies that had been threatened by another dog were slightly more likely to display aggression towards strangers as adults [41], potentially highlighting a sensitivity to traumatic events involving other dogs experienced at a young age. It seems reasonable to suggest that pleasant experiences with calm dogs may be beneficial but unpleasant experiences with dogs may be deleterious to the future behaviour of the dog.

In this regard, O’Sullivan et al. [30] noted that dogs that had been involved in more than one biting incident and not socialised with children were over 8 times more likely to display HDA. This was the only paper in the current study to consider children separately, although there is considerable research examining dog aggression towards children [63,64]. Various programmes have been designed to improve the safety of children around dogs [65] and to educate parents on safe child–dog interactions [66]. However, the precise role of socialisation, specifically with children, is unclear, particularly as the majority of bites to younger children are from familiar dogs in the home environment [52]. Despite the number of programmes designed to reduce bites inflicted on children, follow-up evaluation research on such interventions is generally lacking [67].

The way the dog is managed and trained in the early months of life may also provide useful information about the risk of HDA, but even greater caution is required in accepting this evidence, as discussed below. O’Sullivan et al. [30] noted that dogs that were fed from the table in the two months preceding the biting incident or subjected to verbal or physical punishment were more likely to have a history of repeated aggression. Le Brech et al. [40] also found a significant association between feeding from the table and HDA, although the width of the confidence intervals in both studies suggests that caution should be exercised in evaluating the importance of this. Inconsistency in training methods was linked to higher levels of aggression when compared to dogs that had been trained using consistent methods [37], even if that involved consistent use of punishment. It could be that feeding from the table reflects inconsistent behavioural reinforcement—for example, sometimes fed table scraps but sometimes told off for begging [68]. Feeding from the table might therefore be an indirect indicator of more general problems with owner interaction consistency, which can be expected to result in increased environmental uncertainty and aggression by a dog as a result. 

Blackwell et al. [37] was the only paper to specifically examine the effects of different training methods, i.e., using positive punishment or positive reinforcement. Other authors have focused instead on the effect of attending training classes compared to no attendance at training classes or training carried out at home, compared to in a class. For example, González-Martínez et al. [44] compared dogs that had attended puppy classes based on positive reinforcement training methods with dogs that had not attended any classes and reported no difference in terms of HDA. Dogs who had not attended classes were more than twice as likely to display aggressive behaviour towards other dogs than those who had attended classes. As noted, this might indirectly affect the risk of HDA, as owners often intervene and risk redirected aggression in such circumstances. Regardless of whether training was in a class setting, private training with a professional or undertaken at home by the owner, greater responsiveness to owners’ cues was associated with less aggression for basic behaviours such as sit, down, come and heel [36]. Lack of response to the ‘sit’ command was also shown to be linked to a higher likelihood of aggression [30]. The author notes that in isolation, such a lack of compliance to simple training cues may not be important. However, when considered in the broader context of being fed from the table and possibly subjected to punishment, this might suggest an inconsistent style of dog–owner interaction that may contribute towards the development of HDA rather than specific training effects. Some research [69,70] has claimed that training based on positive reinforcement improves the dog–owner relationship more than the use of aversive training methods. However, any form of effective training will likely involve some form of consistent dog–owner communication and thus potentially increase the dog’s autonomy in the environment and reduce frustration. Inconsistent treatment could thus potentially be a risk factor for the development of HDA through effects on frustration, and frustration has been implicated as a factor in the development of aggressive behaviour [71]. 

As well as a response to frustration, aggression may also be a response to threatening, i.e., fearful stimuli [59,72]. Socialisation and training of puppies are typically aimed at producing a confident and friendly dog. When a young dog experiences distressing events or is not socialised appropriately, then there is an increased risk of developing fear responses and displays of aggressive behaviour as a result. Indeed, O’Sullivan et al. [30] found that dogs displaying fearful reactions were four times more likely to display HDA than non-fearful dogs. The function of aggressive behaviour is often to avoid close proximity to a fear-inducing stimulus. Appleby et al. [24] considered avoidance behaviour and aggression and noted that 68.2% of the dogs showing avoidance behaviour were from a non-domestic maternal environment. Dogs from non-domestic maternal environments were also over-represented in the figures for aggression towards unfamiliar people (both within or away from home), with 66.8% being from a non-domestic maternal environment. Excluding the dogs that showed avoidance behaviour, 62.0% of dogs showing aggressive behaviour were from a non-domestic maternal environment, indicating a link between avoidance and aggressive behaviour. Fear can arise from an encounter with something that the dog has previously found to be unpleasant or from something with which the dog has no prior experience [73]. Inadequate socialisation/habituation may therefore lead to high levels of novelty in the environment, which can increase the risk of aggressive responses based on fear/anxiety. The link between fear and aggressive behaviour in dogs is well documented, and more recent research corroborates the findings of the current study by determining that aggression frequently stems from fear [74]. This may particularly be the case if the dog is restrained, such as being on a leash, and therefore has no escape/flight option. 

Overall, the data suggest that early experiences, which reduce frustration and/or fear, may be particularly important in reducing the risk of HDA in later life. 

### 4.3. Early Litter Experience, including Composition

Some papers highlighted the importance of elements outside the direct control of prospective owners and not directly related to experience. Although genetic factors were not the focus of this study, Reisner et al. [36] found that dogs with a particular pedigree were, on average, 1.6 times more likely to display HDA than dogs of the same breed that were not descendants of this lineage. However, it cannot be assumed that this is necessarily the result of a genetic factor, as there may be early maternal-related influences associated with this factor. For example, the puppies of aggressive mothers may be exposed to more aggression prior to weaning. Temperament is the product of both genetics and early experience. The parents’ temperaments may affect the risk of future HDA, but it remains uncertain to what degree this effect is genetic rather than experiential. Cultural factors related to the owner and their tolerance/acceptance of aggression may also play a role in shaping early temperament; for example, certain types of individuals may be attracted to certain breeds but also encourage aggressivity in their dogs [75]. As the focus of the current work was the effect of early life experiences on the behaviour of the adult dog, the issue of breed-related aggression will not be considered further.

The sex ratio of the litter (presence of more males than females) was also reported to be significant in the risk of development of had [38] in adult dogs. Studies in other mammals have shown that females lying between two males in utero may be exposed to increased testosterone levels before birth, which results in altered hormone levels and reproductive organ development alongside changes in aggressivity (see Ryan and Vandenbergh [76] for a review). This has been proposed to also be relevant to dogs [77]; however, dogs have a zonal placenta, so the chances of exchange between foetuses would appear to be much less likely. 

### 4.4. Factors Found Not to Be Significant

Bearing in mind the caveats already mentioned about the differences in quality, methodology and use of control groups, it is useful to consider those factors which have been examined but which researchers have failed to find statistically significant.

Although it was found that homing outside the 6–8 week window may predispose the dog to developing HDA, it is worth noting that no significant associations between homing age and HDA to familiar people were found by Jokinen et al. [42], although adverse reactions to unfamiliar people were associated with homing age. In contrast, Le Brech et al. [40] did find that early homing (birth to 7 weeks) was associated with a seven-fold increase in the likelihood of aggressive behaviour. These contrasting findings may be due to differing methodologies. Jokinen et al. [42] investigated dogs in Finland, where puppies are normally reared in the owner’s home and purposefully excluded dogs that had not been raised in that way, whereas Le Brech et al. [40] examined dogs in Spain with no information about the source of the dog or the environment prior to rehoming. It may be that the more relevant factor here is not the homing age but the early experiences of the puppy prior to rehoming, i.e., being raised in a home environment predisposes the puppy to interact in a non-aggressive fashion with care givers.

Neither parity, birthweight, growth weight, nor litter size were found to be a factor in HDA by Foyer et al. [31]. This was the only paper to consider these factors, and so caution is required before dismissing their relevance entirely. The dogs in this study were also part of the Swedish Armed Forces Working Dog Programme and cannot, therefore, be said to be representative of the typical pet dog [34]. For the working dogs in this study, aggression could be deemed to be an appropriate response in certain circumstances, such as bite work—although arguably, an appropriately trained dog will not be displaying true aggression in these situations but instead responding to a well-understood and trained cue. The behavioural tests used depicted experiences (e.g., exposure to gunfire) that would be unlikely to be encountered by a pet dog.

Professionals who work with aggressive dogs may point to other factors that have not been the subject of the scientific research presented here. The lack of evidence in this review should, therefore, not be taken to mean that a risk factor is not genuine. For example, nutrition (quality and quantity) may be considered important, as well as overall health, neither of which have been mentioned in this paper, despite being factors that are applicable to the study of puppies.

## 5. Conclusions

This paper has critically reviewed the available scientific literature concerning how the early life experiences of the dog may impact the risk of occurrence of HDA in the adult animal, with a view to making the best use of the available information. Definitions of aggression vary, and it is important to give careful consideration to the implications of the nature of the groups chosen to serve as controls. Three broad areas of risk were identified: acquisition, management and husbandry, and litter-related factors. Despite issues with the quality of individual studies, the synthesis and analysis of the data support the view that early life experiences are important to this risk, probably through the development of temperaments associated with fearfulness and poor frustration tolerance. However, more work is required to establish exactly what factors within these broad areas are most important. It is also apparent that several areas have either not been considered or would benefit from further research, e.g., the health of the dog [46,47]. Recommendations to help standardise the reporting of risk factors for HDA, which will enable greater synthesis and comparison of studies in future, are given in the accompanying box (Box 1).

Box 1 **Recommendations for the standardisation of reporting of risk related to human-directed aggression by dogs**

1. **Inclusion of a clear definition of the form of aggression being studied**, e.g., the risk of a bite versus the risk of growling and biting. Currently, many researchers offer no clear definition;
2. **A clear distinction between the risk factors relating to the occurrence of an inci-dent versus the severity of an incident**. It is often unclear whether researchers are referring to the likelihood or severity of an incident, and some studies appear to combine both;
3. **Definition of the target of the aggressive behaviour**. Risk factors may vary with different human demographics (e.g., family members versus strangers, children versus adults);
4. **Identification and specification of the control group**. Although finding an ideal control group may be difficult, the limits and implications of the chosen control group need to be clearly considered and described;
5. **Detail effect sizes/odds ratios**. When considering risk factors, an indication of the size of the risk is often more valuable than its statistical significance. Inspection of confidence limits will often allow practical translation and evaluation of statistical value;
6. **Acknowledgement of the limitations from sample differences when a compari-son is made with other studies**. Human-directed aggression in dogs is a complex and multi-factorial topic; it is unlikely that two groups of researchers will study identical populations, and the importance of this should be made explicit. Howev-er, the consistency of effect across various studies should be noted;
7. **Avoidance of confirmation bias in the interpretation of risk factors**. It is important to consider the complex nature of many risk factors and not assume what might appear to be the most overt element of a given risk, e.g., that temperament relates to genetics or that socio-economic status relates to the level of care given to the dog.

It is recognised that this paper has concentrated solely on one set of historical risk factors which could contribute towards the risk of HDA, and the importance of these compared to more proximate factors like the day-to-day management of the dog and immediate antecedents to an incident (e.g., actions of owner and victim and broader context), deserves further evaluation. Nonetheless, there is sufficient evidence to suggest positive management practices that might help reduce the risk: in short, it is suggested animals be brought up in environments where they have had a variety of experiences that enable them to develop confidence and cope with frustration and where interactions with humans are consistent and positive.

## Figures and Tables

**Table 1:** Papers considering early life experiences on human-directed aggression in the adult dog.

Paper No.	Authors	Date	Paper Title	Definition of Aggression	Control Group
1	Martin [35]	2001	Is there a correlation between puppy socialization classes and owner-perceived frequency of behaviour problems in dogs?	Provided body language descriptions starting with tense body and escalating up to biting	Littermates that had not attended puppy classes
2 *	Appleby et al. [24]	2002	Relationship between aggressive and avoidance behaviour by dogs and their experience in the first six months of life	Growl, bite, snap, lunge, snarl	Dogs who had behavioural problems associated with lack of control and attention seeking
3	Reisner et al. [36]	2005	National survey of owner-directed aggression in English Springer Spaniels	Towards owners—growl, snap or bite Towards strangers—bite	N/A
4	Blackwell et al. [37]	2008	The relationship between training methods and the occurrence of behaviour problems, as reported by owners, in a population of domestic dogs	No definition given	N/A
5 *	O’Sullivan, et al. [30]	2008	The management and behavioural history of 100 dogs reported for biting a person	Bite, defined as a visible mark left on the victim’s skin	Dogs who had only bitten once (accidental/uncharacteristic)
6	Foyer et al. [38]	2013	Early experiences modulate stress coping in a population of German shepherd dogs	Growl, bite, snap, lunge, snarl	N/A
7 *	McMillan et al. [39]	2013	Differences in behavioral characteristics between dogs obtained as puppies from pet stores and those obtained from non-commercial breeders	Used C-BARQ questionnaire scenario ratings	Non-commercial breeders
8	Foyer, et al. [31]	2016	Levels of maternal care in dogs affect adult offspring temperament	Growl, bite, snap, lunge, snarl	N/A
9	Le Brech, et al. [40]	2016	Canine aggression toward family members in Spain: Clinical presentations and related factors	Growl, bite, snap, lunge, snarl	Aggression to strangers in control group (but not to owners)
10	Pirrone et al. [33]	2016	Owner-reported aggressive behavior towards familiar people may be a more prominent occurrence in pet shop-traded dogs	Growl, bite, snap, lunge, snarl	Official breeder (no definition of ‘official’)
11	Serpell and Duffy [41]	2016	Aspects of Juvenile and Adolescent environment predict aggression and fear in 12-month-old guide dogs	Growl, bite, snap, lunge, snarl	None or N/A
12	Jokinen et al. [42]	2017	Homing age influences the prevalence of aggressive and avoidance-related behaviour in adult dogs	Growl, snap, bite, or attempt to bite	Different homing ages: 6–7 weeks 8 weeks 9–12 weeks 13–16 weeks
13	Marion et al. [43]	2018	Study of aggressiveness in livestock-guarding dogs based on rearing method	Barking, lunging or biting	Dogs reared with some human contact
14	González-Martínez, et al. [44]	2019	Association between puppy classes and adulthood behavior of the dog	Used C-BARQ questionnaire scenario ratings	Dogs that had not attended puppy classes

Paper numbers are subsequently used in data summaries to reduce unnecessary text. * Authors state that the dogs were adults but not explicitly that they were 12 months old or more.

**Table 2:** Developmental risk factors for HDA towards all humans (known and unknown).

Specific Risk Factor (Independent Variable)	Reference Variable	OR	CI
From a rescue (5) *	Acquired from breeder or private owner	“dogs were equally likely to have been obtained from a breeder, a private owner or a rescue facility with 34% having been obtained from a rescue facility”	Unable to calculate
Not socialised with people, raised in sheepfolds (13) *	Socialised with people, raised partly in households	*OR = 5*; **0.0084**	*0.82–30.46*
Attendance at puppy class (14) *	No puppy class attendance	**Not significant**	
Lower level of maternal care (8) *	Higher level of maternal care	WaldsX^2^(1) = 21.8; **<0.001**	Unable to calculate
Season of birth (6) *		F = 1.99 Litters born Jan–March had higher aggression scores than those born Oct–Dec; **<0.1**	Not reported. Unable to calculate
Season of birth (8) *		Wald’s x2(3) = 15 Litters born during summer months scored more highly for aggression; **0.002**	Not reported. Unable to calculate
Training methods used (9) *	Trained using methods other than positive reinforcement only/not trained Trained using methods other than positive reinforcement and consistent punishment/not trained Trained using methods other than positive reinforcement and inconsistent punishment/not trained	Positive reinforcement only *OR = 0.2*; **>0.01** Positive reinforcement and consistent punishment *OR = 0.7*; **>0.01** Positive reinforcement and inconsistent punishment *OR = 1.85*; **>0.01**	*0.04–0.95* *0.3–1.6* *0.79–4.34*

Average effect size/odds ratio (OR) calculated using mean unless otherwise specified. Italics indicate author’s own calculations made from information available in published paper. Statistical interpretation in bold. Confidence interval (CI) at 95%. * Paper number from Table 1.

**Table 3:** Developmental risk factors for HDA towards the owner.

Specific Risk Factor (Independent Variable)	Reference Variable	OR	CI
Sourced from a pet shop (10) *	Acquired from a breeder	OR = 2.4; **0.009**	1.23–4.68
Availability advertised—from a newspaper advert or posted advert (3) *	Other sources of advertisement (word of mouth/contacting a breeder)	OR = 1.7 **not provided**	1.3–2.1
Sourced from a hobby breeder (3) *	Other sources (home-bred, professional kennel, other)	OR = 1.3 **not provided**	1.03–1.7
Dog acquired for the purposes of hunting (3) *	Any other reason for acquisition (guard dog, pet, etc.)	OR = 0.6; **<0.001**	0.5–0.8
Owner has previous experience owning or fostering puppies (11) *	No puppies raised	Owner raised 1 or 2 previous puppies OR = 0.47; **0.0002** Owner raised 3 or 4 previous puppies OR = 0.61; **0.078** Owner raised 5+ previous puppies OR = 0.29; **<0.0001**	0.32–0.70 0.35–1.05 0.16–0.44
Not growing up with other dogs (11) *	Growing up with other dogs in the home	OR = 0.35; **<0.0001**	0.23–0.54
Socialised with people in puppy class (14) *	Not attending puppy class	“No relationship was found between attending puppy classes and owner-directed aggression”	0.09–0.27
Feeding dog from table (9) *	Not feeding from table	OR = 5.86; **0.003**	1.83–20.75
Sleeping in owner’s bed (3) *	Not sleeping in owner’s bed	**No significant association with HDA to owner** (figures not provided)	
No training (3) *	Dogs that had received training	**No significant association with HDA to owner** (figures not provided)	

Average effect size/odds ratio (OR) calculated using mean unless otherwise specified. Italics indicate author’s own calculations made from information available in published paper. Statistical interpretation in bold. Confidence interval (CI) at 95%. * Paper number from Table 1.

**Table 4:** Developmental risk factors for HDA towards family members and familiar people.

Specific Risk Factor (Independent Variable)	Reference Variable	OR	CI
From a non-domestic maternal environment (2) *	From a domestic environment (living in residential part of breeder’s home)	*OR = 1.43*; **0.1** (excluding dogs that showed avoidance and aggression towards unfamiliar people)	*0.85–2.39*
Dogs adopted/rehomed after 7 weeks of age (9) *	Dogs adopted/rehomed from birth to 7 weeks of age	OR = 7.08; **0.04**	1.58–40.32
Trained in class (4) * Trained using no positive punishment	Trained only at home Use of positive punishment	No OR reported; insufficient data available to calculate **<0.05** No OR reported; insufficient data available to calculate **<0.01**.	Not reported, unable to calculate

Average effect size/odds ratio (OR) calculated using mean unless otherwise specified. Italics indicate author’s own calculations made from information available in published paper. Statistical interpretation in bold. Confidence interval (CI) at 95%. * Paper number from Table 1.

**Table 5:** Developmental risk factors for HDA towards strangers and unfamiliar people.

Specific Risk Factor (Independent Variable)	Reference Variable	OR	CI
From a non-domestic maternal environment (2) *	From a domestic environment (living in residential part of breeder’s home)	*OR = 17.86*; **0.02** (Only away from home)	*5.97–53.38*
		*OR = 2.94*; **0.01** (Only at home)	*1.34–6.42*
		*OR = 1.71*; **0.06** (Both at and away from home)	*0.89–3.29*
		*OR = 1.09*; **0.5** (Excluding dogs that showed avoidance and aggressive behaviour towards unfamiliar people)	*0.62–1.92*
Homing age (12) *	Homed at 6–7 weeks At home Outside	*OR = 1.06*; **not statistically significant in pairwise calculations** *OR = 0.19*; **not statistically significant in pairwise calculations**	*0.77–1.47* *0.14–0.26*
	Homed at 8 weeks At home Outside	*OR = 0.86*; **not statistically significant in pairwise calculations** *OR = 6.35*; **not statistically significant in pairwise calculations**	*0.63–1.17* *5.09–7.93*
	Homed at 9–12 weeks At home Outside	*OR = 0.88*; **not statistically significant in pairwise calculations** *OR = 0.32*; **not statistically significant in pairwise calculations**	*0.61–1.27* *0.23–0.43*
	Homed at 13–16 weeks At home Outside	*OR = 2.21*; **0.005** *OR = 0.71*; **0.005**	*1.29–3.80* *0.43–1.16*
Owner has previous experience owning or fostering puppies (11) *	No previous puppies raised	Owner raised 1 or 2 previous puppies OR = 0.92; **0.65** Owner raised 3 or 4 previous puppies OR = 0.99; **0.97** Owner raised 5+ previous puppies OR = 0.51; **0.001**	0.63–1.33 0.60–1.64 0.34–0.77
Socialised with people in puppy class (14) *	Not attending puppy class	“No relationship was found between attending puppy classes and stranger directed aggression”	0.24–0.46
Attendance at puppy class (1) *	Not attending puppy class	Frequency of aggression during play behaviour Unable to calculate OR; **0.05** Frequency of aggression when subject to verbal/physical discipline Unable to calculate OR **0.05**	Not reported. Unable to calculate
Experience of traumatic event (threatened by a dog) (11) *	No experience of traumatic event	OR = 1.16; **0.004**	1.16–2.34
Feeding dog from table in the 2 months prior to bite incident (5) *	Not feeding from table	OR = 3; **0.04**	1–8
Use of verbal/physical punishment on dogs with previous history of aggression (5) *	Use of physical/verbal punishment on dogs with no previous history of aggression	OR = 5; **0.015**	1.4–18
Dog with previous history of aggression being allowed to win tug games (5) *	Dog with no previous history of aggression being allowed to win tug games	OR = 3; **0.019**	1–8
Dogs with previous aggression history allowed to initiate play (5) *	Dogs with no previous aggression history being allowed to initiate play	OR = 3; **0.02**	1–9
Dogs with previous history of aggression not responding to ‘sit’ command (5) *	Dogs with no previous history of aggression not responding to ‘sit’ command	OR = 0.2; **0.015**	0.05–0.7
Dog with previous history of aggression—obedience varied according to location (5) *	Dog with no previous history of aggression—obedience varied according to location	OR = 3; **0.037**	1–8
Dog with previous history of aggression—obedience varied according to person (5) *	Dog with no previous history of aggression—obedience varied according to person	OR = 4.21; **0.005**	1.54 ^+^
Dogs with history of aggression displayed fearful reactions in specific contexts (5) *	Dogs with no previous history of aggression displaying fear in specific context	OR = 5; **0.004**	1.6–15
Dog with previous history of aggression displaying problem behaviours when family members present (5) *	Dog with no previous history of aggression displaying problem behaviours when family members present	OR = 6: **0.02**	1–28
Dog with previous history of aggression displaying 3 or more excessive behaviours (5) *	Dog with no previous history of aggression displaying 3 or more excessive behaviours	OR = 4; **0.028**	1–16
Dog with previous history of aggression not trusted with children (5) *	Dog with no previous history of aggression not trusted with children	OR = 6.1; **0.003**	1.9–20

Average effect size/odds ratio (OR) calculated using mean unless otherwise specified. Italics indicate author’s own calculations made from information available in published paper. Statistical interpretation in bold. Confidence interval (CI) at 95%. * Paper number from Table 1. ^+^ as reported in article, original author unable to explain anomalous figure.

## Data Availability

Data sharing is not applicable. No new data were created or analysed in this study. Data sharing is not applicable to this article.

## Supplementary Materials

The following supporting information can be downloaded at https://www.mdpi.com/article/10.3390/ani13142329/s1: Figure S1: Preferred Reporting Items for Systematic Reviews and Meta-Analysis (PRISMA) flow chart completed for the current study.
