# Supplementary material for: An Assessment of Scientific Evidence Relating to the Effect of Early Experience on the Risk of Human-Directed Aggression by Adult Dogs
Source: Animals (Basel). 2023 Jul 17;13(14):2329. doi: 10.3390/ani13142329 (PMC10376210; doi:10.3390/ani13142329)
Supplement: Supplementary file 1 [file animals-13-02329-s001.zip › animals-2490707-supplementary.pdf]

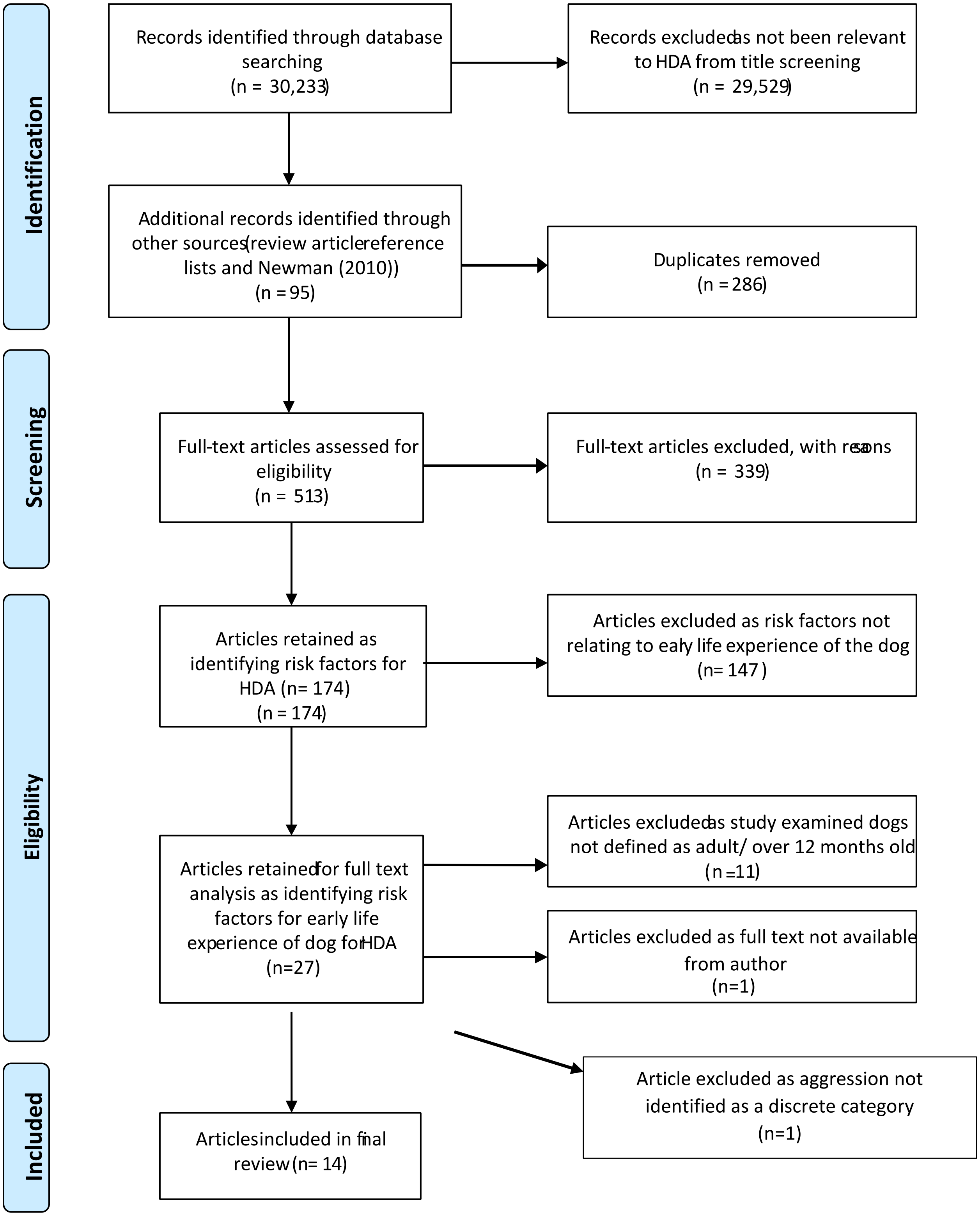

Figure S1. Preferred Reporting Items for Systematic Reviews and Meta-Analysis (PRISMA) flow chart completed for the current study.
